# Breast Cancer and p16: Role in Proliferation, Malignant Transformation and Progression

**DOI:** 10.3390/healthcare9091240

**Published:** 2021-09-21

**Authors:** Dalibor V. Jovanovic, Slobodanka L. Mitrovic, Milos Z. Milosavljevic, Milena B. Ilic, Vesna D. Stankovic, Milena S. Vuletic, Milica N. Dimitrijevic Stojanovic, Danijela B. Milosev, Goran L. Azanjac, Vladica M. Nedeljkovic, Dragce Radovanovic

**Affiliations:** 1 Department of Pathology, Faculty of Medical Sciences, University of Kragujevac, 34000 Kragujevac, Serbia; dalekg84@medf.kg.ac.rs (D.V.J.); lena.ilic@medf.kg.ac.rs (M.B.I.); wesna.stankovic@medf.kg.ac.rs (V.D.S.); milena.vuletic@medf.kg.ac.rs (M.S.V.); milicadimitrijevic@medf.kg.ac.rs (M.N.D.S.); 2 Department of Pathology, University Medical Centre Kragujevac, 34000 Kragujevac, Serbia; m.milosavljevic77@gmail.com (M.Z.M.); danijelamilosevkg@gmail.com (D.B.M.); 3 Department of Plastic Surgery, University Medical Centre Kragujevac, 34000 Kragujevac, Serbia; gazanjac@gmail.com; 4 Institute of Pathology, Faculty of Medicine, University in Pristina—Kosovska Mitrovica,38220 Kosovska Mitrovica, Serbia; vladica.nedeljkovic@med.pr.ac.rs; 5 Department of Surgery, Faculty of Medical Sciences, University of Kragujevac, 34000 Kragujevac, Serbia; drakce_5@hotmail.com

**Keywords:** breast cancer, p16 (INK4A) protein, cellular senescence, molecular subtype, immunohistochemistry

## Abstract

The definition of new molecular biomarkers could provide a more reliable approach in predicting the prognosis of invasive breast cancers (IBC). The aim of this study is to analyze the expression of p16 protein in IBC, as well as its participation in malignant transformation. The study included 147 patients diagnosed with IBC. The presence of non-invasive lesions (NIL) was noted in each IBC and surrounding tissue. p16 expression was determined by reading the percentage of nuclear and/or cytoplasmic expression in epithelial cells of IBC and NIL, but also in stromal fibroblasts. Results showed that expression of p16 increases with the progression of cytological changes in the epithelium; it is significantly higher in IBC compared to NIL (*p* < 0.0005). Cytoplasmic p16 expression is more prevalent in IBC (76.6%), as opposed to nuclear staining, which is characteristic of most NIL (21.1%). There is a difference in p16 expression between different molecular subtypes of IBC (*p* = 0.025). In the group of p16 positive tumors, pronounced mononuclear infiltrates (*p* = 0.047) and increased expression of p16 in stromal fibroblasts (*p* = 0.044) were noted. In conclusion, p16 protein plays an important role in proliferation, malignant transformation, as well as in progression from NIL to IBC.

## 1. Introduction

Invasive breast cancer (IBC) is a heterogeneous group of tumors that show different biological behavior, prognosis, and response to treatment [1]. Numerous studies have investigated the association of individual biomarkers with clinicopathological characteristics and/or treatment outcomes [2,3,4]. Despite the significance of prognostic factors, their accuracy in assessing disease outcomes and determining therapeutic strategies is limited. Therefore, the definition and validation of new molecular biomarkers could provide a more reliable approach in predicting the prognosis of this disease, which would indicate the possibility of application for therapeutic purposes.

Literature data indicate the importance of cell cycle dysregulation in malignant transformation. Cell cycle regulation is controlled by a number of factors, of which p16 protein is one of the most important. p16 (p16^INK4a^ or cyclin-dependent kinase 2A inhibitor) is a protein that blocks cell cycle progression from G1 to S phase by inhibiting the activity of cyclin-dependent kinase complexes 4 and 6 (CDK4/6)/cyclin D1 (CCND1) [5,6]. CCND1/CDK4/6 normally phosphorylates retinoblastoma protein (pRb), its inhibition results in a hypophosphorylated form of pRb, which binds E2F transcription factors and leads to cell cycle arrest and transcription inhibition [7,8,9].

Constant activation of p16, which is most often the result of DNA damage, leads to permanent cell cycle arrest and senescence [10,11,12]. Cell senescence is an irreversible cell cycle arrest in the G0 phase, resulting from telomere shortening or due to oncogenic stress, without the possibility DNA synthesis after mitogenic stimuli [13]. The irreversibility of senescence suggests the assumption that it could be one of the factors that suppress malignant transformation and tumor progression [14]. One of the senescence forms can be started with oncogene activation, which results in the so-called oncogene-induced senescence (OIS). It results in the activation of a number of antioncogenes, which are responsible for the introduction and maintenance of the cell in the G0 phase. One of the most important molecules of OIS is p16, which is considered a tumor suppressor due to its physiological role and reports on its role in the origin and development of IBC are contradictory [15,16,17,18,19,20,21].

The aim of this study is to analyze the expression of p16 protein in IBC, as well as its participation in malignant transformation.

## 2. Materials and Methods

### 2.1. Study Design

The study, approved by the decision of the Ethics Committee number 01/17/2290, included 147 patients with a diagnosis of breast cancer, who were diagnosed and treated at the University Clinical Center in Kragujevac, Serbia in the period from 2012 to 2017.

Pathohistological analysis of tumors on H&E stained specimens was performed on the operative material obtained by tumerectomy, quadrantectomy and/or mastectomy with dissection of regional lymph nodes [22]. The presence of non-invasive lesions (NIL) [in situ lobular and ductal carcinoma (ISC), lobular and ductal atypical hyperplasia (AH) and normal ductal and acinar epithelium (NE)] was noted in each IBC and surrounding tissue. Based on microscopic analysis, IBCs were classified into three groups: ductal, lobular, and a group of other histological types. E-cadherin expression differentiated ductal from lobular breast cancer. At the same time, all relevant macroscopic, pathohistological and prognostic parameters (tumor size, histological type and grade, nodal status, presence of necrosis, intra and peritumoral mononuclear infiltrate, perineural, lymphatic and vascular invasion, molecular subtype IBC and disease stage) were defined [23].

IBCs are classified according to the recommendations into four molecular groups: Luminal A, Luminal B, HER2+, and TNBC [24].

### 2.2. Immunohistochemical (IHC) Procedure

Tissue sections were fixed in 10% neutral buffered and stabilized formalin solution, pH 7.0 and embedded into paraffin. IHC was performed on a tissue section of a representative paraffin block of each patient. Tissue sections 4 μm thick were applied to adherent slides (SuperFrost^®^ Plus, VWR, Leuven, Belgium), then deparaffinized in xylene and rehydrated in decreasing alcohol concentrations. After epitope retrieval, endogenous peroxidase was blocked with 3% hydrogen peroxide for 5 min. The preparations were incubated with primary monoclonal and polyclonal antibodies at room temperature for the recommended duration. The following antibodies were used, ready for use or in appropriate dilution: p16^INK4a^ antibody (CINtec Histology Kit, ROCHE, Mannheim, Germany), mAb ER (1D5, RTU, IR657, DAKO, Gloustrup, Denmark), mAb PR (PgR636, RTU, IR068, DAKO, Gloustrup, Denmark), pAb HER2 (1:1200, AO485, DAKO, Gloustrup, Denmark), Ki67 (1:200, MIB-1, IR626, DAKO, Gloustrup, Denmark). After washing the primary antibody, tissue sections were incubated with commercial biotinized secondary antibody at room temperature, for the recommended duration (En Vision FLEX HRP, RTU, K8000). The IHC reaction was visualized using 3,3′-diaminobenzidine tetrahydrochloride (DAB). The preparations were finally contrasted with Mayer’s hematoxylin (Hematoxylin M, HEMM-OT-1L, Biognost, Croatia), and the coverslips were mounted with Canada balsam. Tissue samples in which incubation with the primary antibody was omitted were used as negative controls of the IHC reaction, and IBCs with known expression of the analyzed markers were used as positive controls. Slides were analyzed at 100×, 200×, and 400× magnifications, using a light microscope (AxioScop 40, Carl Zeiss, Germany). Representative sections were photographed using a digital camera (AxioCam ICc1, Carl Zeiss, Germany).

### 2.3. Evaluation of IHC Staining

Immunohistochemical stains were rated by two independent pathologists, who were blinded to clinical follow-up data at the time of analysis. In cases of differently assessed expression, agreement was reached by joint analysis of the preparation and consultation with a third pathologist.

Analysis of estrogen (ER) and progesterone receptors (PR) expression was performed using Allred score [25] as the sum of the percentage of positive nuclei of tumor cells and the intensity of IHC staining. The Allred score ranges from 0 to 8.

The expression of human epidermal growth factor receptor 2 (HER2) was analyzed based on standard recommendations [26]. Depending on the continuity and intensity of membrane staining, all IBCs were classified into HER2 negative (0 and 1+) and HER2 positive (3+). Equivocal HER2 (2+) was retested by silver in situ hybridization (SISH) technique after which patients were classified as HER2 positive or negative IBCs.

Ki67 IHC expression was defined as the percentage of positive tumor cells per 100 counted in the zone of highest tumor proliferation. According to the previously defined limit value of Ki67 expression in our laboratory, IBCs were classified into 3 groups: low (Ki67 < 15%), medium (Ki67: 15–30%), and high proliferative activity (Ki67 > 30%) [24,27,28].

p16 expression was determined as the percentage of nuclear and/or cytoplasmic expression in IBC and NIL epithelial cells, but also in stromal fibroblasts. By analyzing the expression, we defined the cutoff value for p16. Based on the obtained result, we divided all IBCs into p16 positive and p16 negative. At the same time, p16 expression was analyzed in stromal fibroblasts and classified into: negative (<10%), low (10–24%), medium (25–50%), high (>50% positive cells) [29]. Presence of intra and peritumor mononuclear infiltrate was defined as: absent (0%), low (<30%), medium (30–60%), and high (>60%) [30].

### 2.4. Statistical Data Processing

The commercial software package SPSS (version 22.0, SRSS Inc., Chicago, IL, USA) was used for statistical processing of the data obtained. In the analysis of the obtained results we used: descriptive statistics methods, Man–Whitney test, Kruskal–Wallis test, χ2 test, Pearson or Spearman correlation coefficient, ROC curve (with determination of cutoff value, sensitivity and specificity). By determining the sensitivity and specificity of the test, the level of practical reliability of statistical analysis was determined. All reported *p* values were 2-sided and *p* < 0.05 was considered statistically significant.

## 3. Results

### 3.1. General Characteristics

Our study included 147 women with IBC, with an average age of 58 years (range 29–84). Simultaneously with IBC, ISC (ductal or lobular) was present in 79 patients, in 82 AH, and in 109 cases NE were noted. The average cancer size was 22.5 mm (range 9–68 mm). Clinicopathological characteristics are summarized in Table 1.

### 3.2. p16 Expression Increases with the Progression of Cytological Changes in the Epithelium

**Figure 1:**
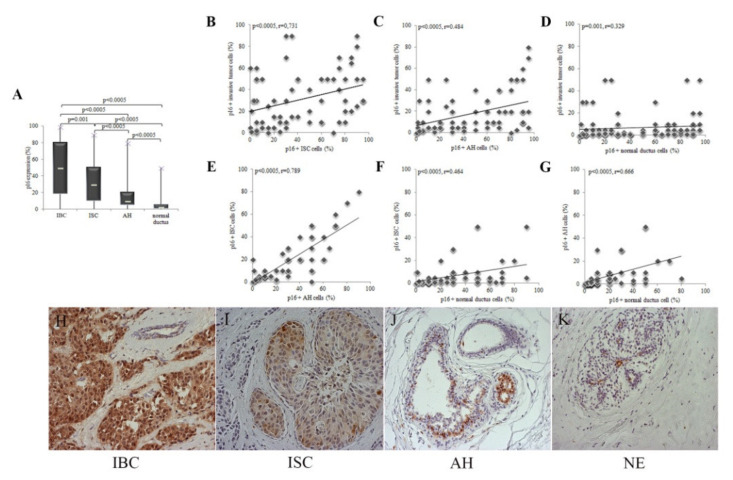
Patients were divided into four groups according to the type of change in the breast: with IBC (*n* = 147), ISC (*n* = 79), AH (*n* = 82), and NE (*n* = 109). IHC expression of p16 was quantified in the nucleus and cytoplasm of tumor cells and presented as a percentage of positive in relation to the total number of evaluated cells. (**A**) The expression of p16 is significantly higher in IBC compared to NIL (*p* < 0.0005). The result is shown as the median. Statistical significance was analyzed using the Kruskal–Wallis and Mann–Whitney tests. (**B**–**D**) The increase in expression in IBC was accompanied by an increase in expression in ISC, AH, and NE. (**E**,**F**) Increased p16 expression in ISC was accompanied by increased expression in AH and NE. (**G**) Increased expression of p16 in AH is accompanied by an increase in expression in NE. Statistical significance was analyzed using the Spearman correlation coefficient. IHC expression of p16 in various lesions in the breast: (**H**) IBC. (**I**) ISC. (**J**) AH. (**K**) NE. (original magnification 200×).

The average value of p16 expression increases from NE, through AH and ISC to IBC and is in NE 2.5%, in AH 10%, in ISC 30%, and in IBC 50%. The expression of p16 is significantly higher in IBC compared to NIL (Figure 1A). Furthermore, p16 expression in IBC cells increases with expression of the same marker in NIL cells. Using the Spearman correlation coefficient, we found a strong positive correlation between p16 expressions in all of these changes (Figure 1B–G). The histomorphological picture of various changes with p16 expression is shown in Figure 1H–K.

### 3.3. The Expression of p16 in Tumor Cells Depend on the Molecular Subtype of IBC

IBCs are classified into molecular subtypes in accordance with the recommendations [24]. Their representation in our sample is shown in Table 2.

**Figure 2:**
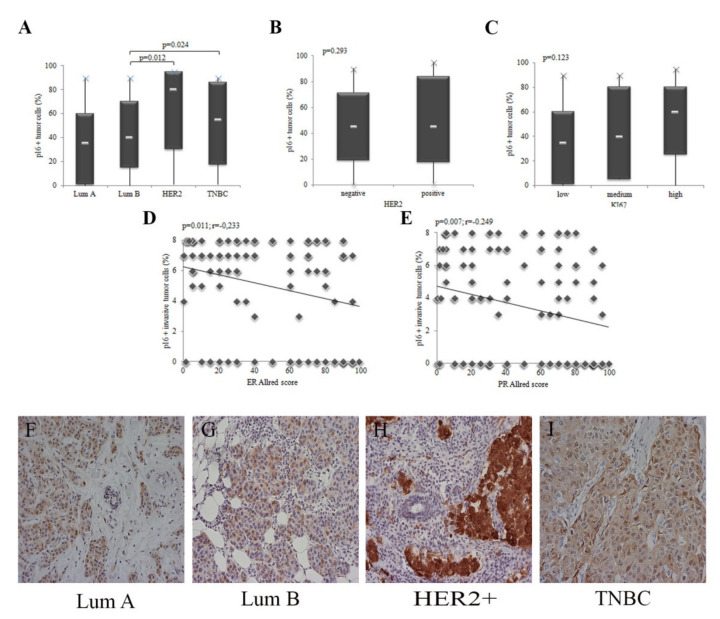
Expression of p16 in different molecular subtypes of breast cancer. All IBCs are divided into four molecular subtypes. IHC expression of p16 was quantified in the nucleus and cytoplasm of tumor cells and presented as a percentage of positive in relation to the total number of evaluated cells. (**A**) HER2+ subtype IBC showed the highest expression of p16. The result is shown as the median. Statistical significance was analyzed using the Kruskal–Wallis and Mann–Whitney tests (*p* = 0.025). (**B**) All IBCs were divided into negative and positive depending on HER2 status. There is no difference in p16 expression depending on HER2 expression. The result is shown as the median. Statistical significance was analyzed using the Mann–Whitney test (*p* = 0.293). (**C**) Ki67 expression is categorized into 3 groups: low (Ki67 < 15%), medium (Ki67: 16–30%) and high (Ki67 > 30%). The expression of p16 in tumor cells does not depend on the expression of Ki67. The result is shown as the median. Statistical significance was analyzed using the Kruskal–Wallis and Mann–Whitney tests (*p* = 0.123). (**D,E**) The ER and PR expression was analyzed through the Allred score. The increase in p16 expression in tumor cells was accompanied by significantly reduced ER and PR expression. Statistical significance was analyzed using by Spearman correlation coefficient (*p* = 0.011; r = −0.233; *p* = 0.007; r = −0.249). IHC expression of p16 in molecular subtypes—(**F**) Luminal A. (**G**) Luminal B. (**H**) HER2+. (**I**) TNBC (original magnification 200×).

There is a difference in p16 expression between different molecular subtypes of IBC (Figure 2A). There is no difference in p16 expression depending on the expression of HER2 and Ki67 (Figure 2B,C). However, with increasing p16 expression in tumor cells, the expression of ER and PR decreases significantly, respectively (Figure 2D,E). The IHC expression of p16 in individual molecular subtypes is shown in Figure 2F–I.

### 3.4. p16 Expression Is a Marker of Breast Tumor Progression

**Figure 3:**
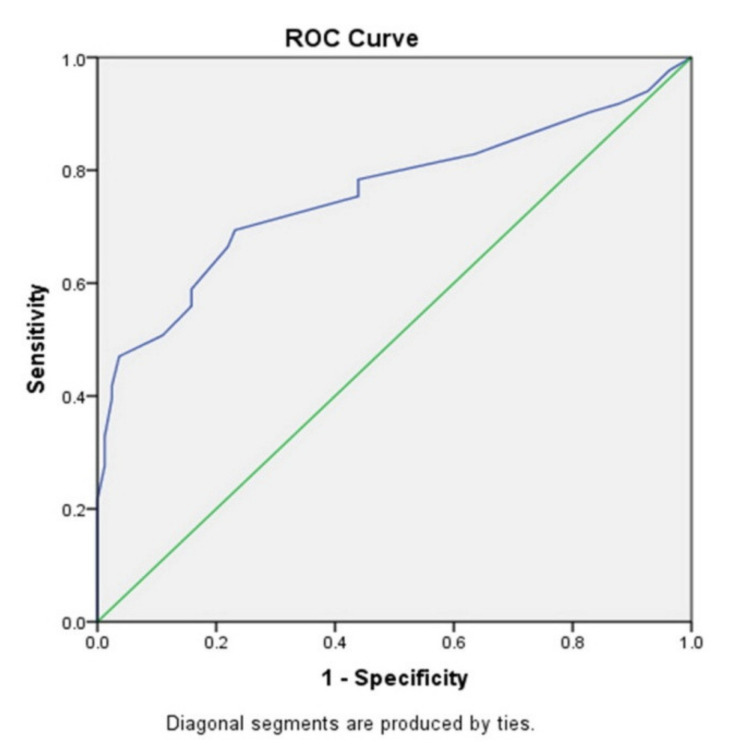
ROC curve of p16 expression in NIL and IBC.

As presented in Figure 3, our results and the obtained ROC (receiver operating characteristic) curve, indicate that increased p16 expression may be a reliable marker of IBC progression (area under the receiver operating characteristic, AUROC = 0.770; *p* < 0.0005).

Also, the results show that a cutoff value of 17.5% of p16 positive tumor cells allows obviously separation of NIL patients from IBC patients (sensitivity 0.754, specificity 0.638). Statistical analysis confirmed that increased p16 expression indicates malignant transformation of lesions in the breast.

**Figure 4:**
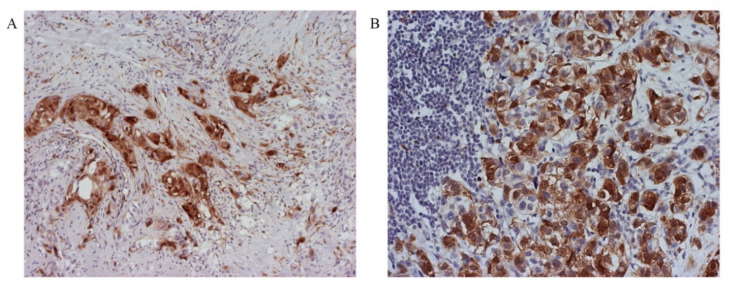
(**A**) p16 overexpression in stromal fibroblasts (original magnification 400×) and (**B**) rich mononuclear infiltrate in p16 positive IBCs (original magnification 400×).

To analyze the association of p16 expression with parameters indicating tumor progression, we applied the cutoff values of our calculation and IBCs were divided into the group with negative (≤17.5%) and positive p16 expression (>17.5%). Further, our study showed that in the group of p16 positive IBCs, increased expression of p16 in stromal fibroblasts was observed (Figure 4A) and increased presence of mononuclear infiltrate (Figure 4B).

Furthermore, we found an association between p16 positive IBCs and examined clinicopathological characteristics (Table 3).

### 3.5. Tumor Invasiveness Depends on the Subcellular Localization of p16 Protein Expression

Cytoplasmic expression of p16 may indicate tumor progression, as opposed to nuclear staining, which is characteristic of most noninvasive changes (Table 4).

**Figure 5:**
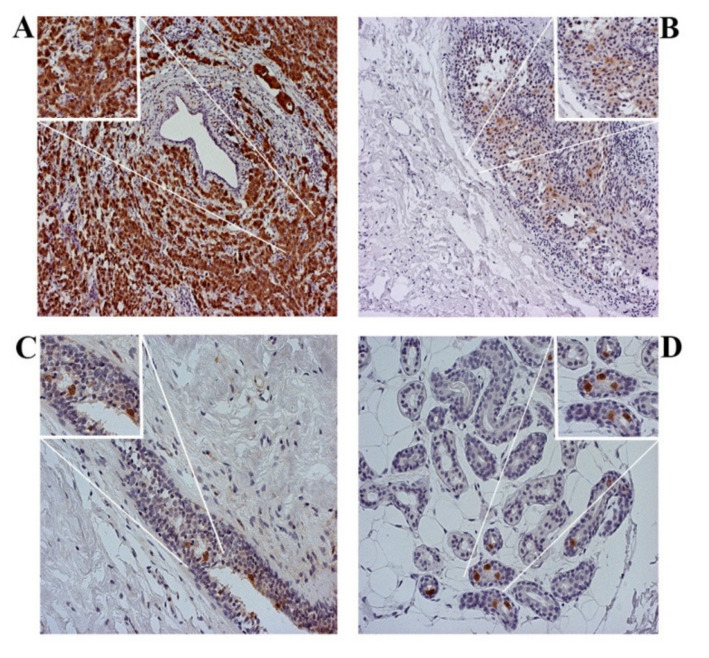
p16 IHC, different subcellular localizations. (**A**) IBC—dominant cytoplasmic staining. (**B**) ISC—dominant cytoplasmic staining, with individual nuclear staining. (**C**) AH—dominant nuclear staining and individual cytoplasmic staining. (**D**) NE—nuclear staining (original magnification 200×).

Dominant nuclear expression of p16 present in NE and AH, in contrast to cytoplasmic expression in ISC and IBC where it was highest (Figure 5).

The percentage of tumors with cytoplasmic expression of p16 was significantly higher in the group of tumors with necrosis present and pronounced mononuclear infiltration (Table 5). In addition, a higher degree of p16 positive fibroblasts was found in the same group of tumors, although the difference was not statistically significant (Table 5). Furthermore, the subcellular localization of p16 was not associated with other clinicopathological parameters (results not shown).

## 4. Discussion

Molecular profiling has provided evidence of breast tumor heterogeneity, and there is therefore a continuing interest in identifying new markers that will help predict prognosis and response to therapy.

In our study, we examined the expression of p16 protein in the tissue of 147 patients with IBC in whose environment the fields of ISC, AH, and normal ductoacinar structures of the breast parenchyma are present. The results of the study showed that the expression increases from NE, through AH and ISC to IBC. Consistent with the fact that p16 tumor is a suppressor protein, its expression increases with increasing epithelial cell proliferation, as a natural response of tumor suppressor to resist uncontrolled growth. In such circumstances, expression should reach its maximum in ISC changes, followed by a plateau in IBC, indicating a role for p16 in preventing malignant transformation of preinvasive changes in the breast [31].

Benign tumors overexpress p16, which inhibits cell proliferation in response to oncogenic stimuli, protecting cells from malignant transformation. However, by activating alternative signaling pathways, p16-dependent tumor suppression, in some cases, will not exhibit adequate function resulting in uncontrolled cell proliferation [32,33]. Based on this, p16 expression may have diagnostic significance in the differentiation of premalignant and malignant lesions [34]. OIS has a strong antitumor role, which has been shown in various benign lesions such as nevus, neurofibroma, and schwannoma, and is accompanied by overexpression of p16 and cell cycle arrest [35,36,37,38,39,40]. In contrast, malignant forms of these tumors show weak or completely absent p16 immunoreactivity which is associated with malignant transformation and progression [38,39,40,41,42].

The results of our study show significantly higher p16 expression in the IBC group than in NIL. A progressive increase in p16 expression from normal to neoplastic tissue has been described in a number of different malignancies [43,44,45,46,47,48,49]. A similar pattern has been observed in skin where p16 expression increases from low levels in premalignant lesions (actinic keratosis) to high levels of expression in in situ and invasive cancers [50]. In addition, increased nuclear expression of p16 has been shown in premalignant and malignant gallbladder lesions, compared with normal epithelium [51]. By studying cytological changes in the squamous epithelium of the cervix, a significantly higher expression of p16 in invasive carcinoma was demonstrated compared to expression in low and high squamous intraepithelial dysplastics lesions, and it was suggested that this marker could be used to differentiate noninvasive from invasive cervical changes [52,53].

Rahmawati Pare et al. show higher expression of p16 in invasive cancer compared to NIL in the breast [31]. The results of this and other studies are consistent with ours, and show that increased p16 expression in malignant breast tissue correlates with negative ER and PR status [31,44,54,55]. In addition, we indicate that increased p16 expression is accompanied by an increased proliferation index determined by Ki67 nuclear expression, as reported by other authors [56,57]. These results indirectly show that high p16 expression assumes a poorer prognosis of the disease [56,57]. The p16 protein is present in the G1 phase of the cell cycle and has a very long half-life, which is why it accumulates in cells with an increase in the number of divisions. This may partly explain the higher p16 expression in highly proliferative tumor cells than in normal breast epithelial cells, and may also be the reason for the positive correlation between p16 expression and Ki67. Gauthier et al. have shown that women with a pathohistological diagnosis of ISC who express high p16 and high Ki67 develop a new tumor, and accordingly Ki67 classifies high p16 expression into two groups: those who develop subsequent breast cancer and another group of women who do not. Tumors that develop after ISC with high p16/high Ki67 expression are often invasive breast cancer. The remaining ISCs showing high p16 immunopositivity had low Ki67, indicating that these cells retained regulation of the p16/Rb checkpoint. Indeed, most lesions exhibiting a high p16/low Ki67 expression phenotype were not associated with ISC recurrence or IBC occurrence [58]. In the Kerlikowske trial, p16 was the only individual marker correlating with an invasive recurrence after ISC [59]. 

Our analysis of molecular types of IBC showed that HER2+ and TNBC have the highest expression of p16. Shin et al. reported an association of high p16 expression with Luminal B and HER2+ tumors [60] while other studies presented results similar to ours, particularly regarding the association of high p16 expression in TNBC [61,62,63,64].

In different benign and malignant changes, it has been observed that p16 expression may occur in the nucleus and/or cytoplasm simultaneously, suggesting that different localization of p16 has a different role in the process of proliferation and tumorigenesis [65]. By analyzing the results of our study, we observed an increasing trend of epithelial cytoplasmic expression of p16 from NE, via AH and ISC to IBC, where expression was also the highest, in contrast to nuclear expression which was highest in NE and progressively decreased towards IBC. Numerous studies have attempted to explain the importance of different subcellular localization of the p16 protein. The regulation of the cell cycle, as the most important function of p16, takes place in the nucleus. On the other hand, cytoplasmic staining in some tumors indicated tumor progression and poor prognosis [66]. For example, in breast tumors, p16 expression was limited to the nucleus in fibroadenoma, and nuclear-cytoplasmic or exclusively cytoplasmic expression was observed in cancer [44,45]. Cytoplasmic expression of p16 can be explained by the formation of the p16 complex with sequestered CDK4, which cannot pass through the nuclear membrane and consequently accumulates in the cytoplasm [46,67,68]. Furthermore, its localization may depend on post-translational modifications or its ability to form a complex with other proteins [69]. p16 interacts with various proteins in the cytoplasm, e.g., with α-β-γ actin and α-β tubulin which play a role in cell migration and cell cytoskeleton construction [70]. Another protein associated with aberrant accumulation of p16 in the cytoplasm is anion exchanger 1 (AE1) [71]. The interaction of transmembrane protein AE1 and p16 in gastric and colon carcinomas results in sequestration of p16 and leads to the accumulation of both proteins in the cytoplasm [71]. In addition, Shen et al. show that decreased AE1 function induces the release and transition of p16 from the cytoplasm to the nucleus, leading to cell death and inhibition of tumor growth [71]. Because of all of the above, localization of p16 in the cytoplasm may represent an alternative mechanism for modulating different signaling pathways, rather than a simple way to inactivate the cell cycle control function [67].

A significant result of our study is that in p16-positive IBCs, there is a more pronounced intra and peritumor mononuclear infiltrate, as well as higher p16 expression in stromal fibroblasts. A group of authors examined p16 expression in the stroma of cervical mucinous carcinoma and concluded that overexpression of p16 in stromal cells contributes to the growth, progression, and more aggressive behavior of this tumor [72]. Published results suggest that the p16 protein promotes invasiveness through interactions with other molecules associated with tumor cell migration [67,73,74,75,76]. Overexpression of p16 in tumor cells and stromal fibroblasts contributes to tumor progression by secreting proinflammatory cytokines (IL-1, IL-6 and IL-8) and proteases (e.g., matrix metalloproteinases) [77,78,79]. Prolonged presence of such cells maintains a chronic inflammatory microenvironment that is essentially tumorigenic [80,81,82,83]. Further, it modulates other important features of cancer by local facilitation of neovascularization [84], epithelial-mesenchymal transition [80,85], tumor invasion [86,87], and cell plasticity [88]. p16-positive stromal fibroblasts produce molecules that can promote tumor development in vivo and malignant phenotype formation in cell culture models. These effects have been observed in a large number of cell types, including breast tumor cell lines [80,89,90,91,92], skin [93], prostate [80,94], pancreas [95], and oro-pharyngeal mucosa [96]. One of the most immediate, protumorogenic effects is to promote epithelial cell proliferation. In the case of breast parenchyma, stromal fibroblasts stimulate the proliferation of premalignant and malignant epithelial cells [80,89,91]. It has also been shown that human lung stromal fibroblasts can promote tumor growth, stimulating angiogenesis by secreting increased concentrations of proangiogenic factors, one of which is vascular endothelial growth factor [84]. All of the above indicates that p16 expression in stromal fibroblasts and more pronounced mononuclear infiltrate affect the growth and progression of various neoplasms, including IBC.

Regarding standard biomarkers in all cases of IBC, hormonal status (ER and PR), Ki-67 proliferative index and HER2 are determined, in order to define the prognosis and establish therapeutic possibilities, including hormone therapy, chemotherapy, and anti-HER2 therapy. However, unlike p16, these markers cannot tell us the difference between NIL and IBC. In the Kerlikowske trial, p16 was the only individual marker correlating with an invasive recurrence after ISC [59]. Special importance should be given to TNBC tumors due to the lack of targeted therapy [97]. Earlier studies performed on the animal model IBC and on material of human origin confirmed an increase in p16 expression in the TNBC group, in particular, basal-like tumor subtypes. Standard markers for determining the basal type phenotype are EGFR, CK5/6 and CK14, and these studies have shown their association with p16 expression [63,98,99].

In this way, the importance of p16 expression in the group of basal-like breast cancer was confirmed. However, the disadvantage of these studies is the reading of p16 expression in the nucleus and cytoplasm, which we tried to compensate by a special analysis of the subcellular localization of p16. Our study opens the door to future research, where special attention would be paid to the association of subcellular localization of p16 and TNBC.

## 5. Conclusions

Our results confirmed that increased p16 expression indicates malignant transformation of changes in the breast and progression of IBC. A cutoff value of p16-positive tumor cells allows the separation of patients with NIL from those with IBC, so that IHC analysis of p16 expression can be used as an additional diagnostic test in separating benign from malignant changes in the breast. Also, excessive cytoplasmic and loss of nuclear expression of p16 are very important in the evolution of tumors from NE to IBC and indicate tumor progression. All of the above could recommend this molecular marker for use for diagnostic purposes or as a therapeutic target.

## Figures and Tables

**Table 1:** Clinicopathological characteristics of IBC.

Variables		N	%
Side	left	66	44.9
	right	81	55.1
Histological type	lobular	18	12.4
	ductal	123	84.8
	other	4	2.8
Histological grade	HG1	17	11.9
	HG2	73	51
	HG3	53	37.1
Nuclear grade	NG1	17	15.2
	NG2	64	57.1
	NG3	31	27.7
Tumor necrosis	absent	26	21.7
	present	94	78.3
Perineural invasion	absent	101	68.7
	present	46	31.3
Lymphatic invasion	absent	72	48.9
	present	75	51.1
Vascular invasion	absent	113	76.9
	present	34	23.1
Molecular subtypes	Luminal A	30	20.4
	Luminal B	76	51.7
	HER2+	19	12.9
	TNBC	22	15
HER2	negative	115	79.3
	positive	30	20.7
Ki67	low	30	20.9
	medium	42	29.4
	high	71	49.7
T status	T1	48	35.8
	T2	64	47.8
	T3	9	6.7
	T4	13	9.7
N status	N0	50	37.3
	N1	48	35.8
	N2	19	14.2
	N3	17	12.7

**Table 2:** Frequency of IBC molecular subtypes.

Molecular Subtypes	N	%
Luminal A	30	20.40
Luminal B	76	51.70
HER2+	19	12.93
TNBC	22	14.97
Total	147	100.0

**Table 3:** Association between p16 expression in IBC and examined clinicopathological characteristics.

Variables		p16 Cut off 17.5		Chi-Square	df	*P*
		−	+			
Mononuclear infiltrate	absent	2 (10%)	2 (2.7%)	7.959	3	0.047
	low	13 (65%)	30 (41.1%)			
	medium	5 (25%)	30 (41.1%)			
	high	0 (0%)	11 (15.1%)			
Stromal fibroblasts	absent	2 (9.1%)	2 (3.2%)	8.120	3	0.044
	low	7 (31.8%)	32 (50.8%)			
	medium	3 (13.6%)	17 (27%)			
	high	10 (45.5%)	12 (19%)			
Histological type	lobular	4 (13.8%)	6 (6.7%)	1.410	2	0.494
	ductal	24 (82.8%)	80 (89.9%)			
	other	1 (3.4%)	3 (3.4%)			
Histological grade	HG1	2 (8.3%)	8 (10.5%)	2.709	2	0.258
	HG2	15 (62.5%)	33 (43.4%)			
	HG3	7 (29.2%)	35 (46.1%)			
Nuclear grade	NG1	1 (4.8%)	11 (14.8%)	2.468	2	0.291
	NG2	15 (71.4%)	40 (54.1%)			
	NG3	5 (23.8%)	23 (31.1%)			
Tumor necrosis	absent	6 (23,1%)	20 (21.3%)	0.000	1	1.000
	present	20 (76,9%)	74 (78.7%)			
Perineural invasion	absent	18 (60%)	65 (72.2%)	1.055	1	0.304
	present	12 (40%)	25 (27.8%)			
Lymphatic invasion	absent	10 (33.3%)	45 (50%)	1.891	1	0.169
	present	20 (66.7%)	45 (50%)			
Vascular invasion	absent	23 (76.7%)	66 (73.3%)	0.014	1	0.904
	present	7 (23.3%)	24 (26.7%)			
Molecular subtypes	Luminal A	4 (14.3%)	11 (13.8%)	3.029	3	0.387
	Luminal B	19 (67.9%)	41 (51.2%)			
	HER2+	2 (7.1%)	11 (13.8%)			
	TNBC	3 (10.7%)	17 (21.2%)			
HER2	negative	24 (82.8%)	67 (75.3%)	0.334	1	0.563
	positive	5 (17.2%)	22 (24.7%)			
Ki67	low	4 (16.7%)	11 (15.3%)	7.217	2	0.027
	medium	11 (45.8%)	14 (19.4%)			
	high	9 (37.5%)	47 (65.3%)			
T status	T1	6 (22.2%)	30 (37%)	3.802	3	0.284
	T2	14 (51.9%)	40 (49.4%)			
	T3	3 (11.1%)	3 (3.7%)			
	T4	4 (14.8%)	8 (9.9%)			
N status	N0	6 (33.3%)	24 (40.7%)	0.959	3	0.811
	N1	9 (50%)	22 (37.3%)			
	N2	2 (11.1%)	8 (13.6%)			
	N3	1 (5.6%)	5 (8.4%)			

**Table 4:** Frequency of p16 expression in different subcellular localizations of NIL and IBC epithelial cells.

	p16 Nuclear vs. Cytoplasmic Expression			
	p16 IBC n (%)	p16 ISC n (%)	p16 AH n (%)	p16 NE n (%)
negative	3 (2.3)	3 (3.8)	3 (3.7)	3 (2.8)
nuclear	28 (21.1)	20 (25.6)	38 (46.3)	99 (90.8)
cytoplasmic	102 (76.6)	55 (70.6)	41 (50)	7 (6.4)
Total	133 (100)	78 (100)	82 (100)	109 (100)

**Table 5:** Association of subcellular localization of p16 protein with clinicopathological characteristics of IBC.

Variables		p16 Expression in IBC		Chi-Square	df	*P*
		Nuclear	Cytoplasmic			
Mononuclear infiltrate	absent	1 (5%)	2 (2.9%)	16.236	6	0.013
	low	14 (70%)	26 (37.7%)			
	medium	5 (25%)	30 (43.5%)			
	high	0 (0%)	11 (15.9%)			
Stromal fibroblasts	absent	1 (5%)	2 (3.2%)	11.638	6	0.071
	low	8 (40%)	31 (50%)			
	medium	6 (30%)	13 (21%)			
	high	5 (25%)	16 (25.8%)			
Tumor necrosis	absent	7 (58.3%)	19 (17.6%)	6.609	2	0.037
	present	5 (41.7%)	89 (82.4%)			

## Data Availability

Not applicable.
